# A TR(i)P to Cell Migration: New Roles of TRP Channels in Mechanotransduction and Cancer

**DOI:** 10.3389/fphys.2019.00757

**Published:** 2019-06-18

**Authors:** Jimena Canales, Diego Morales, Constanza Blanco, José Rivas, Nicolás Díaz, Ioannis Angelopoulos, Oscar Cerda

**Affiliations:** ^1^Program of Cellular and Molecular Biology, Institute of Biomedical Sciences, Faculty of Medicine, Universidad de Chile, Santiago, Chile; ^2^Millennium Nucleus of Ion Channels-Associated Diseases, Santiago, Chile; ^3^The Wound Repair, Treatment and Health (WoRTH) Initiative, Santiago, Chile

**Keywords:** TRP channels, mechanotransduction, focal adhesion, actin cytoskeletal remodeling, cancer

## Abstract

Cell migration is a key process in cancer metastasis, allowing malignant cells to spread from the primary tumor to distant organs. At the molecular level, migration is the result of several coordinated events involving mechanical forces and cellular signaling, where the second messenger Ca^2+^ plays a pivotal role. Therefore, elucidating the regulation of intracellular Ca^2+^ levels is key for a complete understanding of the mechanisms controlling cellular migration. In this regard, understanding the function of Transient Receptor Potential (TRP) channels, which are fundamental determinants of Ca^2+^ signaling, is critical to uncovering mechanisms of mechanotransduction during cell migration and, consequently, in pathologies closely linked to it, such as cancer. Here, we review recent studies on the association between TRP channels and migration-related mechanotransduction events, as well as in the involvement of TRP channels in the migration-dependent pathophysiological process of metastasis.

## Introduction

Cells are capable of accurately sensing and effectively responding to diverse stimuli in the external environment (Clapham, 2003). In response to mechanical stimuli, such as fluid shear stress or extracellular matrix stiffness, activation of intracellular signals leads to a robust cellular response in a process referred to as mechanotransduction (Jaalouk and Lammerding, 2009). Mechanotransduction is crucial to numerous physiological processes, including embryonic development and homeostasis of adult organs (Vogel and Sheetz, 2006; Wozniak and Chen, 2009). These processes are the result of diverse cellular responses that together underlie the mechanotransduction events that allow adaptation to physiological demands. Those cellular responses include proliferation, differentiation, survival, death, and migration (Jaalouk and Lammerding, 2009; Sun et al., 2016). Among these processes cell migration is distinct in that mechanical forces play a central role, in both being sensed and forming the response that is generated by the cells, which then promotes the movements needed to carry out a determined physiological function. Recent research has led to the identifcation of a number of novel molecular components of the pathways underlying migration-associated mechanotransduction, helping to elucidate the molecular mechanisms crucial to this important cellular response. Among these, Transient Receptor Potential (TRP) channels have emerged as key players in cell migration (Schwab et al., 2012). A number of studies have defined specific roles for these channels in diverse aspects of mechanotransduction processes (Clapham, 2003; Sharif-Naeini et al., 2008; Yin and Kuebler, 2010; Kuipers et al., 2012; Eijkelkamp et al., 2013). Here, we discuss the role of TRP channels in cell migration from a mechanostransduction perspective, and the specific role they play in the migration-dependent pathophysiological phenomenon of cancer metastasis.

## Mechanotransduction Mechanisms Shaping Cell Migration

Cell migration is a process involved in numerous and diverse important physiological and pathophysiological events, including wound healing, the immune response, embryonic development and metastasis of cancer cells (Locascio and Nieto, 2001; Luster et al., 2005; Bravo-Cordero et al., 2012; Shaw and Martin, 2016). Molecular mechanisms controlling migration are highly dependent on biochemical signals derived from mechanical events, in which the actin cytoskeleton and cell adhesion structures play a key role, since they sense the mechanical forces from the extracellular environment and, in response, generate mechanical forces for migration (Lauffenburger and Horwitz, 1996). Migration is characterized by dynamic cycles consisting of four general steps: protrusion of the leading edge, adhesion to extracellular matrix (ECM), generation of traction forces and detachment of the trailing edge (Lauffenburger and Horwitz, 1996). Migration starts with the polarization and extension of actin-based protrusions, initially filopodium and later lamellipodium, in the direction of migration. In the case of filopodia, actin filaments are organized as long parallel bundles and its formation is dependent on Rho-GTPase Cdc42 activity; conversely, lamellipodia present a branching network of actin filaments and its formation is dependent on the action of Rho-GTPase Rac1 (Hall, 1998; Small et al., 2002; Mattila and Lappalainen, 2008). The forces generated in the edge of lamellipodia by actin assembly are responsible for the formation of protrusions and nascent adhesions at the leading edge of migrating cell (Choi et al., 2008). These nascent adhesions assemble rapidly in response to the attachment of transmembrane receptor integrins to the ECM which subsequently leads to the recruitment of the intracellular adaptor proteins talin and paxillin that link integrins to the F–actin cytoskeleton (Laukaitis et al., 2001; Zaidel-Bar et al., 2003). As the migration process progresses, nascent adhesions can either disassemble or mature into focal complexes and focal adhesions, increasing their size by mechanical tension, and promoting the recruitment of additional proteins (vinculin, alpha-actinin, VASP and Focal Adhesion Kinase or FAK) that generate and stabilize the focal complex, followed by the addition of zyxin and tyrosine phosphatases that lead to formation of focal adhesions (Zaidel-Bar et al., 2003). Mature focal adhesions associate with the end of stress fibers, structures composed of bundles of actin and myosin II that have a high contractile capability (Burridge and Wittchen, 2013). The formation of both stress fibers and focal adhesions are dependent on Rho-GTPase RhoA activity (Ridley and Hall, 1992). The actin-myosin cytoskeleton contraction generates the force necessary to pull the cell body forward (Lauffenburger and Horwitz, 1996). Finally, in the rear of migrating cells, focal adhesions disassemble, promoting cell retraction at the trailing edge, and allowing for forward cellular movement (Ridley et al., 2003).

Ca^2+^ signaling plays a critical role in each of these steps. During cell migration, intracellular Ca^2+^ signaling events are regulated in their amplitude, as well as their spatial and temporal characteristics allowing for an effective control of the consecutive events occurring in the different compartments of the migrating cell (Wei et al., 2012). Ca^2+^ is distributed in a front-to-rear increasing gradient in migrating cells (Brundage et al., 1991; Hahn et al., 1992). In the leading edge of the cell, Ca^2+^ increases occur as “flickers,” directing the cycles of lamellipodia retraction and nascent focal complex formation (Giannone et al., 2007; Wei et al., 2009). Ca^2+^ signaling induces the myosin-light chain (MLC) kinase-dependent phosphorylation of MLC, promoting the contraction of myosin II and the consequent retraction of the actin bundle, including the attachment of the leading edge and stabilization of the nascent focal adhesions (Tsai and Meyer, 2012). In the rear of migrating cells, Ca^2+^ activates the Ca^2+^-dependent protease calpain which catalyzes the cleavage of focal adhesion proteins such as integrins, talin, vinculin and FAK (Glading et al., 2002), promoting the disassembly of these structures and, thereby, allowing trailing edge retraction. Additionally, Ca^2+^/calmodulin activates the serine/threonine phosphatase calcineurin, which mediates the recycling of integrins from the sites of disassembly of trailing edge focal adhesions (Lawson and Maxfield, 1995). Finally, Ca^2+^ acts upstream of cytoskeleton rearrangement and focal adhesion dynamics, modulating the activity of Rho GTPases Rac1 and RhoA, which activates several effectors that promote the lamellipodia formation and regulate the thickness of stress fibers, respectively (Small et al., 2002; Price et al., 2003; Dovas et al., 2006).

As stated above, mechanical forces participate in all steps of migration for effective cell movement. The mechanical forces that are generated in the processes previously described promote successive rounds of cell adhesion, contraction and retraction in response to cellular signals controlling cell movement, such that migration is the net result of several mechanotransduction events. For instance, mechanical stretching of the nascent adhesion protein talin leads to a conformational change in talin that leads to presentation of its cryptic vinculin binding sites (del Rio et al., 2009), leading to the subsequent recruitment of vinculin that is necessary for maturation of nascent adhesions into focal complexes and focal adhesions (Zaidel-Bar et al., 2003). Integrins transmit tension from the actomyosin cytoskeleton to ECM protein fibronectin, producing the conformational changes in fibronectin that are crucial to fibronectin assembly (Zhong et al., 1998). As previously mentioned, each of these molecular events shaping cell migration are controlled by Ca^2+^ signaling. Accordingly, the plasma membrane ion channels mediating Ca^2+^ entry into cells are fundamental components of the mechanotransduction that underlies cell migration. Among these channels, certain members of the TRP channel superfamily play a particularly critical role in mechanotransduction events during cell migration.

## Transient Receptor Potential (TRP) Channels

**FIGURE 1:**
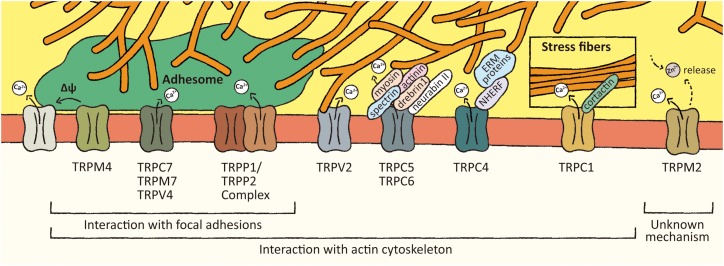
Association of TRP channels with mechanosensitive structures. Several members of TRP channels superfamily interact with focal adhesions and/or actin cytoskeleton-associated proteins. These interactions might regulate TRP channels activity leading to local changes in Ca^2+^ levels and membrane potential. These local effects might promote focal adhesions turnover and actin cytoskeleton remodeling.

TRP channels constitute a superfamily of polymodal non-selective cation channels, formed as tetramers of subunits whose structure is characterized by six transmembrane domains, with the channel pore located between domains 5 and 6, and with cytoplasmic amino (N)- and carboxyl (C)-terminal regions (Nilius and Owsianik, 2011). In mammals, these channels are categorized into six subgroups, based on sequence homology: Canonical TRP channels (TRPC), Melastatin TRP channels (TRPM), Vanilloid TRP channels (TRPV), Mucolipin TRP channels (TRPML), Polycystin TRP channels (TRPP), and Ankyrin TRP channels (TRPA) (Nilius and Owsianik, 2011). TRP channels can be activated by a variety of stimuli, including endogenous and exogenous ligands as well as physical-chemical stimuli, such as temperature, pH changes, tension, osmolarity, and pressure (Clapham, 2003; Nilius and Owsianik, 2011). In general, the mechanosensitivity of plasma membrane ion channel activity has been proposed to be due to either channel gating being controlled by lateral tension at the membrane (the bilayer model) or by a linkage of the channels to ECM and/or subcortical actin cytoskeleton (the tethered model) (Marshall and Lumpkin, 2012). It is presumed that such mechanisms mediate could also allow mechanical changes to regulate TRP channel activity. Indeed, TRP channel activity can be modified by alterations in plasma membrane phospholipids, such as phosphatidylinositol-4,5-biphosphate (PIP_2_) (Nilius et al., 2008), or by changes in cell structural components that alter their gating, such as actin cytoskeleton (Liu and Montell, 2015). Since most TRP channels mediate Ca^2+^ entry, these plasma membrane proteins constitute important candidate molecules controlling cell migration mechanisms from the mechanotransduction perspective. In fact, there is increasing evidence that TRP channels are key regulators of Ca^2+^ signaling mediating the mechanotransduction shaping cell migration, and in particular a role for TRP channels in actin cytoskeleton rearrangement and focal adhesions dynamics (Figure 1).

## Association of TRP Channels With Mechanosensitive Structures

### TRP Channels and the Actin Cytoskeleton

Actin cytoskeleton dynamics are a trascendental aspect of cell migration. These processes control protrusion, adhesion, contraction, and retraction from the cell front to the rear (Gardel et al., 2010). These dynamics are regulated by a variety of actin-associated protein and signaling pathways that are highly dependent on changes in intracellular Ca^2+^ levels, as Ca^2+^ plays a crucial role in the reorganization of the actin cytoskeleton by modulating the function of actin-associated proteins (Pollard and Cooper, 2009; Tang and Gerlach, 2017). Plasma membrane ion channels play a fundamental role in regulating diverse signaling pathways by mediating the flux of ions across the plasma membrane. Among these, the cation permeable TRP channels are especially important in cell migration (Smani et al., 2014). The association of TRP channels with the actin cytoskeleton results in bidirectional communication: on the one hand, TRP channels mediate the flux of ions that promotes actin cytoskeleton reorganization, and conversely, the actin cytoskeleton and associated proteins induce changes in TRP channel location, protein–protein interactions and channel gating, thereby modulating TRP channel function (Smani et al., 2014).

#### Actin Cytoskeleton-Mediated TRP Channel Regulation

Some members of the TRP superfamily directly interact with the actin cytoskeleton, while other members interact indirectly through other associated proteins. For instance, TRPC4, a non-selective cationic Ca^2+^ permeable channel member of the TRP Canonical subfamily. TRPC4 is activated by Gq/phospholipase C-coupled receptors and tyrosine kinases, and has been implicated in endothelial permeability, vasodilation and cell proliferation (Zhu et al., 1996). TRPC4 interacts with NHERF, a molecular scaffold and regulatory factor of the Na^+^/H^+^ exchanger (Tang et al., 2000). NHERF is a two PDZ domain-containing protein associated with the actin cytoskeleton *via* interactions with members of ezrin/radixin/moesin family (ERM) (i.e., ezrin and moesin) through their N-terminal domains (Reczek et al., 1997). The C-termini of ERM proteins interact with F-actin in a manner dependent on oligomerization and ERM phosphorylation states (Tang et al., 2000). Thus, the interaction of TRPC4 with NHERF provides a physical link between these plasma membrane ion channels and the actin cytoskeleton. TRPC4 interacts with NHERF via a PDZ interacting motif present on the C-terminus of TRPC4. The C-terminus of TRPC4 also contains a PIP_2_ binding domain that underlies the PIP_2_-mediated inhibition of TRPC4 activity (Otsuguro et al., 2008). Interestingly, TRPC4/actin cytoskeleton association as mediated by the C-terminal PDZ binding motif is required for PIP_2_-dependent TRPC4 inhibition (Otsuguro et al., 2008). As such, the association of TRPC4 with the ERM/actin cytoskeleton through TRPC4-NHERF interaction regulates PIP_2_-mediated inhibition of TRPC4 activity (Otsuguro et al., 2008). TRPC4 is also associated with to the actin cytoskeleton in endothelial cells, although in this case TRPC4 interacts with the 4.1 protein, which, in turn, is associated with the actin cytoskeleton through spectrin (Cioffi et al., 2003). The association of TRPC4 with the actin cytoskeleton is necessary for TRPC4 to function as a Store Operated Channel (SOC) (Cioffi et al., 2003). These data indicate that the TRPC4/actin cytoskeleton association occurs through different protein complexes, and that this association plays a key role of regulating TRPC4 activity.

TRPC1 is a non-selective cation channel that has also been proposed to be a component of SOC (Rychkov and Barritt, 2007). TRPC1 is also associated with the actin cytoskeleton in myoblasts (Formigli et al., 2009). In this case, TRPC1 can be co-immunoprecipitated with the actin-binding protein cortactin, and this interaction is increased by the sphingosine-1-phosphate-induced formation of stress fibers (Formigli et al., 2009). The cortactin-mediated interaction of TRPC1 with stress fibers is essential for the localization of TRPC1 in cell membrane lipid rafts; such localization is necessary for the TRPC1 channel activity, indicating that in myoblasts TRPC1/actin stress fiber interaction is required for channel activity (Formigli et al., 2009).

Two other members of the Canonical TRP subfamily, TRPC5 and TRPC6, interact with actin cytoskeletal proteins. Immunopurification of TRPC5 and TRPC6 from rat brain lysates and mass spectrometry-based analyses of associated proteins led to identification of a number of cytoskeletal proteins, including spectrin, myosin, actin, actinin, debrin 1 and neurabin II, the latter a F-actin binding protein that has a single PDZ domain (Goel et al., 2005). However, the functional consequences of the association of these TRP channels with these cytoskeletal proteins has not been elucidated.

TRPV4 is a member of the vanilloid subfamily of TRP channels. TRPV4 is a Ca^2+^-permeable TRP channel that was identified as a receptor for a variety of physical and chemical stimuli, such as hypotonicity, moderate heat and synthetic or endogenous agonists (Garcia-Elias et al., 2014). Interestingly, TRPV4 interacts with actin in a number of different cell types (Becker et al., 2009; Goswami et al., 2010). TRPV4 and actin co-localize in highly dynamic pro-migratory membrane structures, such as filopodia and lamellipodia edges (Becker et al., 2009). However, more stable structures, such as F-actin bundles and stress fibers, lack TRPV4. The interaction between TRPV4 and F-actin is required for the activation of these channels by hypotonicity and underlies TRPV4-mediated volume decrease (Becker et al., 2009). TRPV4 also interacts with other cytoskeletal elements including tubulin and neurofilament proteins (Suzuki et al., 2003; Goswami et al., 2010). The TRPV4 C-terminal domain interacts directly with both tubulin and actin, and for both major cytoskeletal proteins in both with their soluble and polymeric forms. Moreover, TRPV4 co-localizes *in vivo* with both actin-based microfilament and tubulin-based microtubule enriched structures at submembrane regions. Elevated expression and/or activation of TRPV4 induces striking morphological changes affecting lamellipodial and filopodial structures (Goswami et al., 2010). There is evidence that TRPV4 preferentially associates with actin filaments in a process that involves phosphorylated Ser824 in its C-terminal tail (Shin et al., 2012). This presumably direct TRPV4-actin interaction promotes TRPV4 cell surface expression, its single channel activity and consequent Ca^2+^ influx (Shin et al., 2012).

Another example of direct interaction of a plasma membrane TRP channel with the actin cytoskeleton is TRPV2, a non-selective cation channel with a relatively high Ca^2+^ permeability, and whose activity is promoted by heat (Shibasaki, 2016). TRPV2 acts as a mechanosensitive ion channel in the digestive tract and during neuronal development (Mihara et al., 2010; Shibasaki et al., 2010). Recently studies show that that local application of mechanical stimuli promotes actin reorganization *via* TRPV2 activation in PC12 cells. TRPV2 physically interacts with actin and actin cytoskeleton changes are required for its activation. Thus the actin cytoskeleton is critical for TRPV2 activation by mechanical stimuli, which subsequently feeds back to impact reorganization of the actin cytoskeleton (Sugio et al., 2017).

#### TRP Channel-Promoted Actin Cytoskeleton Remodeling

The actin cytoskeleton plays a vital role in maintaining the structural integrity of cells and in producing the force necessary to accomplish basic cellular functions, including cytokinesis, contractility, adhesion and migration. Many members of the TRP family act to impact these processes by promoting changes in the actin cytoskeleton. For example, activation of TRPC channels under conditions of hypoxic stress promotes Ca^2+^-dependent MLC phosphorylation, necessary for contraction of actin and myosin filaments, thus generating a contraction of the endothelial cells that disrupts the blood brain barrier, leading to hypoxia-induced increases in its permeability (Hicks et al., 2010).

One of the most common mechanisms by which TRP channels control the actin cytoskeleton is through the activation of Rho GTPases. As mentioned above, Rac1 and Cdc42 play key roles in generating actin-rich lamellipodial and filopodial membrane extensions, respectively. These processes are initiated by peripheral actin polymerization and they are required for both cell spreading and migration (Hall, 1998). Conversely, RhoA is primarily associated with actin stress fiber formation and actomyosin contractility (Ridley and Hall, 1992). TRPC5 and TRPC6 channel activity promotes the activation of Rac1 and RhoA, respectively. TRPC5 suppresses stress fiber and focal adhesion formation through Rac1 activation, promoting a motile phenotype (Tian et al., 2010). Conversely, growth factor-induced Rac1 activation induces TRPC5 membrane translocation, which has been linked to TRPC5 localization in growth cones and filopodia of hippocampal neurons and the subsequent TRPC5-dependent regulation of growth cone motility and morphology (Greka et al., 2003; Bezzerides et al., 2004). Conversely, through RhoA activation TRPC6 activity promotes formation of stress fibers and adhesions resulting in a contractile and stationary cell phenotype (Tian et al., 2010). The mechanisms whereby TRPC6 produces these effects have been studied in a number of different contexts. TRPC6 activity promotes endothelial cell contraction and increases endothelial permeability through enhanced intracellular Ca^2+^ signaling and subsequent PKCα and RhoA activation, which favors MLC phosphorylation and actin stress fiber formation (Singh et al., 2007). In the context of proteinuric kidney disease, TRPC6 overexpression generates RhoA activation and consequent stress fiber formation, thus promoting cell retraction that hinders the extension of foot processes and may lead to proteinuria (Jiang et al., 2011).

TRPV4 function has also been linked to RhoA activity. TRPV4 upregulation promotes actin polymerization and stress fiber formation through RhoA activation in lung fibroblasts derived from patients with idiopatic pulmonary fibrosis, acting as a key mechanosensor for myofibroblast differentiation (Rahaman et al., 2014). Furthermore, TRPV4 participates in the intraocular pressure-induced increase in trabecular meshwork cell stiffness by mediating mechanical stretch-promoted Ca^2+^ entry that is required for cytoskeleton remodeling in the form of enhanced stress fibers (Ryskamp et al., 2016). Conversely, exogenous expression of TRPV4 in breast cancer cells accelerates actin dynamics and is correlated with a higher activation of cofilin, a protein that promotes the severing of actin filaments (Lee et al., 2016).

Numerous TRPM subfamily members have also been linked to cytoskeleton remodeling processes. TRPM4, a Ca^2+^-activated non-selective monovalent cation channel (Vennekens and Nilius, 2007), interacts with diverse proteins involved in actin cytoskeleton dynamics and regulates Ca^2+^ increases, FAK activation and Rac1 GTPase activity. All of these processes impact the actin cytoskeleton reorganization that is involved in cellular attachment and lamellipodia formation, mechanisms underlying both cell spreading and migration (Caceres et al., 2015). Another member of the TRPM subfamily linked to actin cytoskeleton regulation is TRPM7, a TRP channel that is permeable to Na^+^, Mg^2+^ and Ca^2+^, and whose activity is dependent on Mg^2+^ and intracellular ATP levels (Visser et al., 2014). This channel is exceptional in that it contains a C-terminal protein kinase domain (Visser et al., 2014). Knockdown of TRPM7 in fibroblasts leads to a decrease in actin stress fibers and an increase in cortical actin (Su et al., 2011). This correlates with decreased activity of RhoA GTPase, suggesting a role for TRPM7 in RhoA regulation. Additionally, TRPM7 knockdown leads to reduced Rac1 Rho-GTPase activity and alters temporal aspects of Cdc42 Rho-GTPase activation, consequently decreasing lamellipodia formation and impairing the directionality of cell migration (Su et al., 2011).

Beyond TRP channel-mediated Rho GTPase regulation, these ion channels participate in the dynamic modulation of the actin cytoskeleton through Rho GTPase-independent mechanisms. In the context of myoblast migration and differentiation, TRPC1 channel activity is necessary for calpain activation and the consequent calpain-promoted cleavage of actin-binding protein MARCKS (Louis et al., 2008), which has been linked to an increase in actin polymerization rates (Tapp et al., 2005). Conversely, TRPM7 associates with the actomyosin cytoskeleton and, through its C-terminal protein kinase domain, phosphorylates the C-terminal region of myosin IIA heavy chain, which is critical for filament assembly, regulating filament stability and protein localization (Clark et al., 2008). Thus, TRPM7 also participates in Ca^2+^-independent mechanisms for actin-cytoskeleton rearrangement. Interestingly, another member of TRPM channel family, TRPM2, also promotes actin remodeling during cell migration independent of its canonical ion channel activity. TRPM2 is a Ca^2+^ and monovalent cation-permeable ion channel, whose activity is both temperature-sensitive and activated by several ligands and reactive oxygen species (Kashio and Tominaga, 2017). TRPM2 promotes H_2_O_2_-induced filopodia formation in HeLa and PC-3 cells independent of TRPM2-mediated Ca^2+^ entry, but dependent on TRPM2-mediated intracellular Zn^2+^ release (Li et al., 2016). This corroborates the idea that actin cytoskeleton rearrangement mechanisms linked to TRP activity go beyond the canonical pathways involved in Ca^2+^ signaling or Rho GTPases activity. Thus, there remain numerous unexplored aspects of TRP channel function in mechanotransduction during cell migration.

### TRP Channels and Focal Adhesions

Focal adhesions are dynamic structures that connect the F-actin cytoskeleton with the ECM through transmembrane receptor integrins (Singer, 1982; Horwitz et al., 1986; Hynes, 1992; Wehrle-Haller and Imhof, 2002; Winograd-Katz et al., 2014). Focal adhesions contain more than 160 proteins and have pleiotropic functions: adhesion to ECM, binding to actin filaments, cellular signaling and adaptors (Winograd-Katz et al., 2014). A distinct set of Ca^2+^-sensitive proteins, including protein kinases and calpain proteases, regulate the assembly and disassembly of these structures (Giannone et al., 2004; Chan et al., 2010). As such, regulation of Ca^2+^ levels in close proximity to focal adhesions plays an important role in the dynamics of these multiprotein complexes that directly impact cell migration. Growing evidence linking focal adhesions and TRP channels has opened a novel perspective to understand the migration processes.

TRPV4 has been described as a mechanical strain-activated channel, as force applied to β1 integrin promotes rapid TRPV4 activation (Matthews et al., 2010). Moreover, there is evidence supporting a direct role for TRPV4 in focal adhesion dynamics, and particularly on the disassembly of these structures. TRPV4 co-localizes with the focal adhesion marker paxillin, overexpression of a defective form of TRPV4 that is unable to bind PIP_2_ is correlated with lower calpain activity, and cell lines overexpressing TRPV4 show smaller focal adhesions. Conversely, TRPV4 silencing as well as overexpression of the PIP_2_ binding defective TRPV4 mutant results in larger focal adhesions (Mrkonjic et al., 2015). This leads to a model whereby TRPV4 channel regulates local Ca^2+^ levels, which in turn activate protein kinases and calpains involved in the disassembly process (Mrkonjic et al., 2015). A pore-dead TRPV4 mutant mimics the effects on focal adhesions promoted by the non-PIP_2_-interacting TRPV4 mutant, suggesting that TRPV4-mediated Ca^2+^ entry is responsible for all these effects (Mrkonjic et al., 2015).

Certain members of the TRPM and TRPC subfamilies also participate in focal adhesion regulation. TRPM7 regulates focal adhesion disassembly and turnover *via* m-Calpain, by promoting an increase of intracellular Ca^2+^ levels (Su et al., 2006). TRPM4 localizes to focal adhesions where it promotes an increase of intracellular Ca^2+^ levels and induces focal adhesion disassembly *via* regulation of FAK and paxillin activities (Caceres et al., 2015). Similar to TRPM4 channels, TRPC7 localizes to focal adhesions during the Syndecan-4-mediated maintenance of myofibroblast phenotype. Moreover, expression of a non-phosphorylatable TRPC7 mutant (i.e., S714A substitution) leads to aberrant increases in intracellular Ca^2+^ levels and induces the loss of focal adhesions (Gopal et al., 2015).

TRPP1 (also known as Polycystin-1) forms a mechanosensor heterodimer with the six transmembrane domain protein TRPP2 (also known as Polycystin-2) (Retailleau and Duprat, 2014). Within the TRPP1/TRPP2 complex, TRPP1 is proposed to function as a mechanosensor, while TRPP2 acts as the ion channel to mediate Ca^2+^ influx (Retailleau and Duprat, 2014). Consistent with the notion of the TRPP1/TRPP2 heterodimer acts as a mechanosensitive ion channel, TRPP1 is associated with mechanotransductor focal adhesions. Moreover, TRPP1 localizes to focal adhesions and associates with focal adhesion components such as FAK, paxillin and vinculin (Wilson et al., 1999; Geng et al., 2000; Joly et al., 2006). Interestingly, TRPP1 activity has been linked to FAK and paxillin phosphorylation and to adhesion complex formation (Joly et al., 2006). However, whether the proposed role of TRPP1 in regulating focal adhesions is dependent on its heterodimerization with TRPP2 has not been elucidated.

## TRP Channels and Cell Migration

As discussed above, several members of the TRP channel superfamily have been associated with structures and processes fundamental for mechanotransduction in cell migration, in particular the actin cytoskeleton and focal adhesions (Table 1). However, we have not yet addressed the nature and extent of how this translates into a functional role for TRP channels in impacting cell migration.

**TABLE 1:** TRP channels and mechanosensitive structure association: implications for cell behavior.

**TRP channel**	**Mechanosensing structure**	**Effect in TRP channel/ mechanosensing structure**	**Cellular response**	**Model**	**References**
TRPC?	Actin cytoskeleton	Ca^2+^-dependent MLC phosphorylation	Cell contraction	Endothelial cells of blood brain barrier	Hicks et al.,2010
TRPC1	Actin cytoskeleton	TRPC1 localization in lipid rafts	?	Myoblasts	Formigli et al.,2009
	Actin cytoskeleton	Lamellipodia formation	Cell migration	MDCK cell line	Fabian et al.,2008
TRPC4	Actin cytoskeleton	?	?	HEK293 cells over-expressing TRPC4	Tang et al.,2000
	Actin cytoskeleton	PIP2-induced TRPC4 inhibition	?	HEK293 cells over-expressing TRPC4	Otsuguro et al.,2008
	Actin cytoskeleton	Suggested TRPC4 activation	SOC activation	Endothelial cells	Cioffi et al.,2003
TRPC5	Actin cytoskeleton	?	?	Rat brain	Goel et al.,2005
	Actin cytoskeleton	Suppression of fiber stress formation	Cell migration	Ang-ll-treated podocytes	Tian et al.,2010
	Focal adhesion	Suppression of focal adhesions formation	Cell migration	Ang-ll-treated podocytes	Tian et al.,2010
TRPC6	Actin cytoskeleton	?	?	Rat brain	Goel et al.,2005
	Actin cytoskeleton	Fiber stress formation	Cell contraction	Ang-ll-treated podocytes	Tian et al.,2010
	Actin cytoskeleton	Fiber stress formation	Cell contraction	Endothelial cells	Singh et al.,2007
	Actin cytoskeleton	Fiber stress formation	Cell contraction	Podocytes over-expressing TRPC6	Jiang et al.,2011
	Focal adhesion	Focal adhesions formation	Cell contraction	Ang-ll-treated podocytes	Tian et al.,2010
TRPC7	Focal adhesion	Focal adhesion stability	Maintenance myofibroblast phenotype	Myofibroblasts	Gopal et al.,2015
TRPM2	Actin cytoskeleton	Filopodia formation	Cell migration	Hela and PC-3 cell lines	Li et al.,2016
TRPM4	Actin cytoskeleton	Actin reorganization	Cell migration	Fibroblasts	Caceres et al.,2015
	Focal adhesion	Focal adhesions disassembly	Cell migration	Fibroblasts	Caceres et al.,2015
TRPM7	Actin cytoskeleton	Lamellipodia formation	Directional cell migration	Fibroblasts	Su et al.,2011
	Actin cytoskeleton	Actomyosin filaments assembly	?	RBL-2H3 cell line over-expressing TRPM7	Clark et al.,2008
	Focal adhesions	Focal adhesions disassembly	Cell adhesion	HEK293 cells over-expressing TRPM7	Su et al.,2006
TRPV2	Actin cytoskeleton	TRPV2 activation/actin reorganization		PC12 cell line	Sugio et al.,2017
	Actin cytoskeleton	Hypotonicity-induced TRPV4 activation	Volume regulation	CHO cell line over-expressing TRPV4	Becker et al.,2009
	Actin cytoskeleton	TRPV4 membrane localization and activity	Expansion of cell surface area	HEK293 cells over-expressing TRPV4	Shin et al.,2012
TRPV4	Actin cytoskeleton	Filopodia formation	?	F11 cell line	Goswami et al.,2010
	Actin cytoskeleton	Stress fibers formation	Myofibroblast differentiation	Lung fibroblasts	Rahaman et al.,2014
	Actin cytoskeleton	Stress fibers formation	Cell stiffness	Trabecular meshwork cells	Ryskamp et al.,2016
	Actin cytoskeleton	Actin turnover	Endothelial transmigration	Breast cancer metastasis cell lines	Lee et al.,2016
	Focal adhesion	Focal adhesions disassembly	Directional cell migration	HEK293 cells over-expressing TRPV4 and T47D cell line	Mrkonjic et al.,2015
TRPP1	Focal adhesions	?	?	Kidney epithelial cells	Wilson et al., 1999; Geng et al., 2000.
	Focal adhesions	Focal complex formation	Cell spreading	Kidney epithelial cells	Joly et al.,2006

TRPM7 has been linked to several mechanotrasduction mechanisms in cell migration. First, in human embryonic lung fibroblasts, TRPM7 is activated by membrane tension at the leading edge of migrating cells, promoting Ca^2+^ entry, which activates the IP_3_R2 and thereby produces the local Ca^2+^ flickers necessary for cell migration (Wei et al., 2009). TRPM7 overexpression has also been linked to loss of cell adhesion through m-Calpain activation (Su et al., 2006). Accordingly, TRPM7 knockdown induces an increase in the number of focal adhesions and contractility in the breast cancer cell line MDA-MB-231, which correlates with a reduced migratory/invasive phenotype (Middelbeek et al., 2012). The contribution of TRPM7 function to cell migration of human nasopharyngeal carcinoma cells involves Ca^2+^-induced Ca^2+^ release, where TRPM7 activity promotes Ryanodine Receptor activation and, consequently, increases intracellular Ca^2+^ levels and cell migration (Chen et al., 2010). Thus, TRPM7 has been demonstrated to directly impact migration of diverse cell types through its mechanotransduction actions as mediated by intracellular Ca^2+^. TRPM2 channel activity also promotes cell migration, in this case H_2_O_2_-induced migration in HeLa cells (Li et al., 2016). Consistent with the impact on actin remodeling detailed above, the role of TRPM2 in cell migration is independent of the increased Ca^2+^ signals mediated by these channels, but dependent on Zn^2+^ release (Li et al., 2016).

Another member of the TRPM channel subfamily linked to cell migration is the TRPM4 channel. TRPM4 has been implicated in several mechanisms of mechanotransduction in cell migration. Enhanced TRPM4 activity promotes focal adhesion disassembly, actin cytoskeleton reorganization as evaluated through cell spreading, Rho-GTPase Rac1 activation and FAK activation (Caceres et al., 2015). TRPM4 regulates mouse embryonic fibroblast cell migration in a Rac1-dependent manner, and pharmacological TRPM4 inhibition delays skin wound healing *in vivo* (Caceres et al., 2015). Interestingly, TRPM4 inhibition decreases intracellular Ca^2+^ signaling (Caceres et al., 2015). There is increasing evidence that although the TRPM4 channel is not Ca^2+^-permeable, it can act to indirectly regulate intracellular Ca^2+^ levels (Launay et al., 2004; Gonzales et al., 2014). Thus, it is plausible that enhanced activity of TRPM4, through an indirect effect on intracellular Ca^2+^, acts to promotes the processes detailed above that lead to cell migration. TRPV4 channels are also involved in mechanotransduction-promoted cell migration. As detailed above, expression of a mutant TRPV4 defective in PIP_2_ binding inhibits focal adhesion turnover (Mrkonjic et al., 2015). This results in a non-directional migratory pattern, characterized by fast and persistent movements followed by stalling and turning motion (Mrkonjic et al., 2015). Conversely, TRPV4 overexpression leads to a continuous loss of directionality. Thus, it is likely that TRPV4 participates in directionality of migrating cells and formation of trailing edge adhesions, although a causal relationship between both effects has not been clearly established (Mrkonjic et al., 2015). Beyond their effects on focal adhesions, TRPV4 channels have been linked to the regulation of cell stiffness by modulating the actin cytoskeleton. TRPV4 accelerates actin dynamics by promoting F-actin depolymerization, thereby contributing to reduced cell stiffness of breast cancer cells. This feature is essential for endothelial transmigration and extravasation, the process through which metastatic cells traverse the endothelial lining to access the circulatory system and colonize distant organs (Lee et al., 2016). It should be noted that this result is seemingly contradictory to a report that TRPV4 promotes stress fiber formation and cell rigidity in trabecular meshwork cells (Ryskamp et al., 2016). However, the latter study did not address cell migration, but instead the structural stiffness of trabecular meshwork cells in the context of glaucoma disease. It is important to emphasize that to understand the role of TRPV4 in actin cytoskeleton dynamics it is necessary to consider the cell model and the pathophysiological context in which these studies are contextualized.

Another member of the TRPV family associated with cell migration is TRPV2. It has been reported that TRPV2 is activated by the lysophospholipids, lysophosphatidylcholine and lysophosphatidylinositol, and TRPV2-knockdown in the prostate cancer cell line PC3 decreases lysophospholipid-induced migration (Monet et al., 2009). Moreover, exogenous expression of TRPV2 increases migration in the prostate cancer cell line LNCaP (Monet et al., 2010). TRPV2 has been linked to adrenomedullin-promoted adhesion and migration of prostate (PC-3) and urothelial (T24/83) cancer cells in a mechanism involving TRPV2 translocation to the cell membrane and increased cytoplasmic Ca^2+^ levels (Oulidi et al., 2013). However, the role of TRPV2 in regulating specific mechanotransduction mechanisms in cell migration is still undetermined.

Among TRPC subfamily members, TRPC5 activity induces Rac1 activation and subsequent suppression of stress fiber and focal adhesion formation (Tian et al., 2010). Consistent with these mechanistic and structural effects, TRPC5 activity has been linked to higher Angiotensin-I receptor-promoted podocytes migration (Tian et al., 2010). TRPC5-mediated Ca^2+^ signals triggered by sphingosine-1-phosphate in vascular smooth muscle cells have been associated with increased cell migration (Xu et al., 2006), although whether these effects are dependent on the impact of TRPC5 activity to suppress stress fibers and focal adhesions has not been defined. TRPC1-mediated Ca^2+^ entry has been linked to calpain activation in skeletal myoblasts, and this activity is necessary for cell migration during myogenesis *via* a mechanism that presumably involves the calpain-promoted cleavage of the actin-binding protein MARCKS (Louis et al., 2008). Consistent with the impact of TRPC1 on actin dynamics, TRPC1 silencing prevents polarization and yields defective lamellipodia formation and reduced migration in a migrating MDCK model (Fabian et al., 2008).

## TRP Channels and Cell Migration: Implications for Cancer Metastasis

Metastasis is the cause of more than 90% of cancer deaths (Siegel et al., 2011). It is a hallmark of cancer that is characterized by the spread of malignant cells from the primary tumor to distant organs (Talmadge and Fidler, 2010; Hanahan and Weinberg, 2011). In this process, cancer cells from the primary tumor, invade the basal membrane and reach lymphatic and blood vessels. The cancer cells then transit through these systems and extravasate to form distant metastatic foci. Therefore, to metastasize, malignant cells must acquire migration and invasion properties (Friedl and Wolf, 2003). Increasing evidence shows that the stiffness of solid tumors enhances the mechanical forces in tumor cells, leading to the activation of mechanotransduction signals that promote several pro-carcinogenic responses, including cell invasion and metastasis (Kopanska et al., 2016; Wei and Yang, 2016; Broders-Bondon et al., 2018). Thus, the mechanotransduction events occurring during metastasis development provide a new perspective to understand mechanisms involved in cancer progression. As a consequence, the TRP channels that are involved in mechansotransduction are emerging as prominent actors in this context. Some members of the TRP channel superfamily contribute to metastasis, presumably by promoting the cancer cell’s acquisition of a migratory and invasive phenotype, which are fundamental for the development of this advanced stage of the disease.

TRPM7 is the best studied TRP channel in the context of metastasis. In primary breast cancer tumors, higher expression of TRPM7 mRNA correlates with diminished distant metastasis free-survival and free-recurrence survival (Middelbeek et al., 2012). These data are supported by elevated levels of TRPM7 mRNA in metastatic breast cancer compared to primary tumors (Meng et al., 2013). TRPM7 protein is also overexpressed in ductal adenocarcinoma compared to non-tumoral tissue (Guilbert et al., 2009), and TRPM7 levels are increased in invasive areas of Estrogen Receptor negative invasive ductal cancer (Guilbert et al., 2013). TRPM7 silencing in the triple negative breast cancer cells MDA-MB-231 decreased their metastatic potential *in vivo* by reducing their migratory ability, but not their viability (Middelbeek et al., 2012). TRPM7 has also been linked to pancreatic cancer (Yee et al., 2015; Rybarczyk et al., 2017). TRPM7 protein expression correlates with primary tumor size and stage of malignant pancreatic tumors, and is expressed at higher levels in metastatic tumors and in primary metastasizing tumors than normal tissue (Yee et al., 2015). TRPM7 is also overexpressed in pancreatic ductal adenocarcinoma, in which higher TRPM7 levels in primary tumors correlate with higher levels of lymph node metastasis (Rybarczyk et al., 2017). Pancreatic cancer cell lines also exhibit elevated levels of TRPM7 whose silencing decreases their invasive capacity (Yee et al., 2015). TRPM7 is also highly expressed in nasopharyngeal tumors, with higher TRPM7 mRNA levels in metastatic tumors than in primary tumors, and TRPM7 expression is absent in normal nasopharyngeal tissue (Chen et al., 2015). Moreover, elevated TRPM7 expression is associated with poor prognosis and metastasis in this type of cancer (Chen et al., 2015). These clinical data are supported by *in vitro* assays showing that TRPM7 knockdown decreases the migration and invasion of metastatic nasopharyngeal cancer cells, and that TRPM7 overexpression results in increases in both processes in non-metastatic nasopharyngeal cancer cells (Chen et al., 2015). TRPM7 is also associated with bladder cancer, such that TRPM7 mRNA and protein expression are higher in bladder tumor tissue compared to adjacent non-tumoral tissue, and elevated TRPM7 expression correlates with recurrence, metastasis and a poorer prognosis for this disease (Gao et al., 2017). Consistent with this, the TRPM7 knockdown decreases the migration and invasion of bladder cancer cells *in vitro* (Gao et al., 2017). Recently, another TRPM channel subfamily member has been associated to metastatic processes. It has been described that TRPM2 is functionally expressed in gastric cells and that is related to migration and invasion ability of gastric cancer cells *in vitro* as well as to tumor formation capacity and epithelial-mesenchymal transition markers expression *in vivo* (Almasi et al., 2019).

Vanilloid TRP channels subfamily members whose activity has been linked to mechanostransduction and migration have also been associated with cancer metastasis. As such, TRPV4 mRNA expression has been linked to a diminished distant metastasis free-survival in breast cancer samples (Lee et al., 2016). TRPV4 knockdown decreases migration, invasion, and transendothelial migration, but not the viability, of breast cancer cells *in vitro* as well as the number of metastatic lung nodules *in vivo* (Lee et al., 2016). Higher TRPV2 mRNA expression has also been observed in metastatic prostate cancer samples compared to localized prostate cancer samples (Monet et al., 2010). Xenograft tumor*-*based *in vivo* approaches in cancer prostate cells suggest that TRPV2 silencing diminishes the weight of tumors and the expression of invasion markers, namely MMP2, MMP9 and cathepsine B (Monet et al., 2010).

TRPC5 channels have also been linked to cancer metastasis. TRPC5 has been associated with colon cancer progression, and TRPC5 expression is higher in colon cancer samples than in paired normal tissue and also higher in metastatic lymph nodes than in paired primary tumors. Patients with elevated TRPC5 expression patients show a poorer overall survival and free-metastasis survival (Chen et al., 2017a). TRPC5 silencing in highly migratory and invasive colon cancer cells inhibits the migration and invasion, whereas exogenous expression of TRPC5 in colon cancer cells with a weak migration and invasion ability promotes an increase in these parameters (Chen et al., 2017b).

In summary, several members of TRP channel superfamily that have been demonstrated to act in mechanotransduction events translated in a migratory response have also shown to be relevant to metastasis of several types of cancer. These data raise the question whether other TRP channels reviewed here could have a similar pathophysiological potential, which represent a interesting challenge for future research.

## Conclusion

**FIGURE 2:**
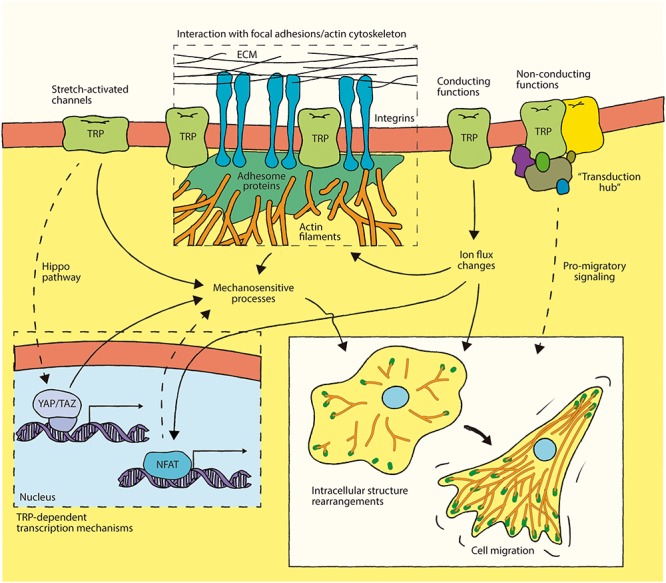
Integration of TRP channel involvement in mechanotransduction-associated mechanisms during cell migration. Some TRP channels interact with proteins at focal adhesions and/or with the actin cytoskeleton. These associations might lead to focal adhesion turnover and actin cytoskeleton rearrangement through a mechanism involving the conducting function of TRP channels, whereby Ca^2+^ influx plays a key role. In addition, non-conducting effects of TRP channels might contribute to regulation of focal adhesions and actin dynamics during cell migration. The putative role of TRP channels as structural component or “transduction hubs” in these mechanosensitive structures appears as a novel perspective to understand the involvement of these channels in mechanotransduction during cell migration. Furthermore, TRP channels could promote pro-migratory long-term effects by gene reprogramming (e.g., Hippo pathway- and/or NFAT activation-mediated), representing a putative new mechanism through which these ion channels could contribute to cell migration.

Increasing evidence demonstrates the role of numerous members of the TRP channel superfamily in mechanotransduction and cell migration. However, in many cases the details of precise role of TRP channels in the underlying mechanisms remain poorly understood. The further dissection of the molecular events regulated by TRP channels constitute an interesting research topic. For example, questions remain as to the specific subcellular localization of these channels during cell migration processes. Moreover, the structural role of TRP channels as molecular platforms for organizing cell signaling complexes and other cellular structures remain largely unexplored. Recently, evidence for non-conducting functions of ion channels support these ideas. For example, the non-conducting or physical contribution of certain ion channels in the maintenance of cellular structures, in this case ER-PM junctions, has recently been demonstrated (Fox et al., 2015; Kirmiz et al., 2018). Thus, ion channels might not only regulate local ion fluxes, but may also serve as “transduction hubs” between plasma membrane structures such as focal adhesions and intracellular compartments. Moreover, since several members of the TRP channel superfamily are stretch-activated, these molecules could contribute to the mechanosensitive-dependent changes of the dynamic structure of these subcellular interactions during cell migration-related processes. Thus, the identification of the TRP channel-associated interactome found at these structures is an intriguing topic to be elucidated. This will provide new insights into the role of these molecules on the regulation of cell migration and mechanotransduction-related processes (Figure 2).

Finally, the role of TRP channels in mechanosensitive-dependent gene expression programs is still unknown. Recent findings have demonstrated the role of different TRP channels in NFAT-dependent transcription mechanisms (Kuwahara et al., 2006; Nakayama et al., 2006; Onohara et al., 2006; Numaga et al., 2010; Weber et al., 2010; Gao et al., 2012). There is also emerging information on the role of the YAP/TAZ transcriptional co-activators related to the Hippo pathway on mechanosensitive-related gene expression programs (Piccolo et al., 2014; Moroishi et al., 2015; Dupont, 2016; Figure 2). As such, the study of the crosstalk between TRP-dependent signaling pathways and these transcriptional mechanisms might contribute to our understanding of the roles of these in channels in long-term modulation of cell migration and mechanotransduction-dependent mechanisms, such as during developmental and differentiation programs, and under pathophysiological conditions, such as cancer.

Consistent with their role in cellular migration, certain TRP channels are associated with progression and metastasis of several types of cancer, likely due to their pro-migratory actions. Therefore, the emerging information on the novel molecular and cellular roles of TRP channels in physiological mechanostransduction and cell migration may also provide fundamental new insights into important pathophysiological processes. Future research in this area could help to clarify the emerging role of TRP channels in cancer metastasis and lead to the identification of novel targets for the development of a battery of new therapeutic alternatives to fight cancer.

## Author Contributions

JC wrote the first draft of the manuscript. JC, DM, CB, JR, ND, IA, and OC wrote sections of the manuscript. OC conceived the idea. All authors contributed to the manuscript revision, read, and approved its final version.

## Conflict of Interest Statement

The authors declare that the research was conducted in the absence of any commercial or financial relationships that could be construed as a potential conflict of interest.
